# Tuberculosis following programmed cell death receptor-1 (PD-1) inhibitor in a patient with non-small cell lung cancer. Case report and literature review

**DOI:** 10.1007/s00262-020-02726-1

**Published:** 2020-10-17

**Authors:** Aasir M. Suliman, Shaza A. Bek, Mohamed S. Elkhatim, Ahmed A. Husain, Ahmad Y. Mismar, M. Z. Sharaf Eldean, Zsolt Lengyel, Shereen Elazzazy, Kakil I. Rasul, Nabil E. Omar

**Affiliations:** 1 Department of internal medicine, Hamad General Hospital, Hamad Medical Corporation, Doha, Qatar; 2 Department of Medical Oncology, National Center for Cancer Care and Research, Hamad Medical Corporation, Doha, Qatar; 3 Department of Infectious Diseases, Communicable Disease Center, Hamad Medical Corporation, Doha, Qatar; 4 Department of Pathology, Hamad General Hospital, Hamad Medical Corporation, Doha, Qatar; 5 Department of Body Imaging, National Centre for Cancer Care and Research, Hamad Medical Corporation, Doha, Qatar; 6 Pharmacy Department, National Centre for Cancer Care and Research, Hamad Medical Corporation, 3050 Doha, Qatar

**Keywords:** Tuberculosis, NSCLC, PD-1 inhibitor, Pembrolizumab, Immune related adverse events, Case report

## Abstract

Immune checkpoint inhibitors (ICIs)—anti-programmed death-1 (PD-1) and their ligands (PD-L1 and PD-L2) have become widely used in the treatment of several malignancies. Many immune-related adverse events (irAEs) have been linked to these agents. Nonetheless, tuberculosis (TB) reactivation during their use is increasingly recognized and reported. Herein, we present a 58-year-old lady with advanced non-small cell lung cancer (NSCLC) ALK-negative, EGFR wild, and PD-L1 immune histochemistry (IHC) strongly positive in 95% of tumor cells, on ongoing treatment with Pembrolizumab as a first-line monotherapy. Our patient presented with 1-week history of productive cough and high-grade fever. Further workup yielded the diagnosis of pulmonary tuberculosis after her Pembrolizumab sixth cycle with positive AFB smear and TB PCR from BAL (rifampin resistance not detected), with negative HIV status. Hence, immunotherapy was held, and patient was commenced on anti-TB regimen. History revealed contact with active TB patient over the past decade, without previous documentation of latent TB or previous TB infection. Her sputum AFB smear remained persistently positive 4 weeks through anti-TB regimen course. Later, the patient was discharged after her sputum was cleared from AFB (two negative sets). In light of pembrolizumab mechanism of action as an immune checkpoint inhibitor, we suspected its implication on reactivating latent TB which was observed in our patient demonstrating features of pulmonary tuberculosis. She was not re-challenged with Pembrolizumab following TB diagnosis.

**Electronic supplementary material:**

The online version of this article (10.1007/s00262-020-02726-1) contains supplementary material, which is available to authorized users.

## Background

Immune checkpoint inhibitors (ICIs) are a type of cancer immunotherapy that has provided a tremendous breakthrough in the field of oncology [1]. They inhibit inhibitory pathways of immune cells which allow more increased immune cell activity and reduce T-cell exhaustion [2]. Currently approved checkpoint inhibitors target the cytotoxic T-lymphocyte-associated protein 4 (CTLA4), programmed death receptor-1 (PD-1), and programmed death-ligand 1(PD-L1).

Recognition of cancer cells by the toxic T lymphocytes plays an essential role in the malignant cell killing. Cancer cells may evade this process by expressing programmed death-ligand 1 (PD-L1), which binds to the programmed death receptor-1 (PD-1) on T-cell surface [3]. This interaction (PD-L1/PD-1) leads to inhibition of cytokines and T-cell proliferation, leading to cancer cells equivocating the killing process. Consequently, immunotherapy-mediating inhibition of (PD-L1/PD-1) pathway has revolutionized cancer treatment [4].

Interestingly enough, the role of ICIs has been studied in infectious diseases as well [5]. Various human studies and animal models suggest that immune system activated by PD-1/PD-L1 blockade is effective in targeting certain viral, bacterial, and fungal pathogens by limiting T-cell dysfunction [5, 6].

Nonetheless, in sharp contrast with other pathogens that cause chronic infection, accumulating reports demonstrate the occurrence of Mycobacterium tuberculosis (MTB) infection during immunotherapy with ICIs (Table 1).

**Table 1:** Summary of case reports documenting development of pulmonary TB in cancer patients treated with immune checkpoint inhibitors

Ref. number	Age/sex	Ethnic origin	Diagnosis	Time to TB diagnosis (after how many cycles of ICPIs	How Diagnosed	Management of TB	Outcome of TB	ICPI resumed
Ref. [10]	50/M	Caucasian	Metastatic melanoma	4 cycles of Pembrolizumab	Histology and tuberculin skin test conversion	4-drug regimen, maintenance of the ICPI	Complete regression of pleural effusion	Yes
	64/M	Caucasian	Metastatic NSCLC	2 cycles of Nivolumab	Histology, positive bone culture and PCR	4-drug regimen, discontinuation of the ICPI	Rapid death after a second operation for spinal cord compression	No
Ref. [15]	59/M	Asian	metastatic NPC	3 cycles of Nivolumab	Histopathology, Positive sputum culture, PCR	discontinuation of the ICPI, 4-drug therapy then IV anti-TB due to patient condition	Expired 1 month after diagnosis with TB reactivation	No
	83/M	Caucasian	metastatic MCC	12 cycles of Pembrolizumab	AFB negative but culture positive, positive IFN-γ release assay	discontinuation of the ICPI, 4-drug therapy, then ICP restarted due to evidence of progression	The patient completed 9 months of TB therapy without evidence of recurrence	Yes
Ref [18]	87/M	Asian	HL	5 cycles of Nivolumab	Positive sputum culture	3-drug regimen, discontinuation of the ICPI	Complete remission of P. TB	No
Ref [19]	72/M	Asian	Metastatic NSCLC	8 cycles of Nivolumab	Positive BAL culture and PCR Positive IGRA conversion	TB therapy (no details given)	Not specified	No details
Ref [20]	59/M	Asian	Metastatic NSCLC	3 cycles of Nivolumab	Histology, and positive pericardial fluid culture	Treatment for TB (no details given), maintenance of the ICPI	Complete regression of pericarditis	Yes
Ref [21]	65/F	Asian	Advanced melanoma	10 cycles of Pembrolizumab	Liquid culture and positive BAL	4-drug regimen and pause of ICPI	Complete remission of P. TB	Yes
Ref [22]	56/M	Caucasian	Metastatic NSCLC	12 cycles of Nivolumab	Histopathology, Positive sputum culture, PCR	Treatment for TB (no details given), discontinuation of the ICPI	Not specified	No
Ref [23]	49/M	Asian	Stage 4 SCC of hard palate	6 cycles of Nivolumab	Positive sputum culture, AFB stain, PCR	Treatment for TB (no details given), discontinuation of the ICPI	The patient expired 5 months after the diagnosis of TB because of bacterial pneumonia with acute respiratory failure	No
Ref [24]	75/M	Asian	Metastatic NSCLC	15 cycles of Nivolumab	AFB stain, Positive sputum culture, PCR	Hold of the ICPI, 4-drug therapy,	Paradoxical response (PR) 10 days after initiation of anti-MTB treatment, culture and AFB negative post 3 months of TB treatment	Yes
Ref [25]	56/F	Caucasian	Metastatic NSCLC	Not defined	AFB stain, positive culture	Discontinuation of the ICPI, 4-drug therapy	Not specified	No
Ref [26]	76/F	Caucasian	Advanced melanoma	8 cycles of Nivolumab +/− Ipilimumab	BAL culture , PCR	Discontinuation of the ICPI, 3-drug therapy	Patient expired with acute respiratory failure 3 days after initiation of Anti-TB	No
	85/M	Caucasian	Metastatic melanoma	9 cycles of Atezolizumab	Sputum Culture	Maintenance of the ICPI, 4-drug therapy	Complete remission of P. TB	Yes
Ref [27]	62/F	Not defined	Metastatic melanoma	Pembrolizumab	Positive BAL	Treatment for TB (no details given)	Clinical improvement, normalization of liver function tests, and regression of the lung lesion	No details
Current case	58/F	Caucasian	Metastatic NSCLC	6 cycles of Pembrolizumab	AFB smear and TB PCR from BAL	Discontinuation of the ICPI, 4-drug therapy	Currently patient is still receiving her Anti-TB medication along with the new line chemotherapy, clinically stable	No

Herein, we present a patient with advanced non-small cell lung cancer (NSCLC) who developed pulmonary tuberculosis following treatment with pembrolizumab monotherapy as first-line treatment.

## Case presentation

**Fig. 1:**
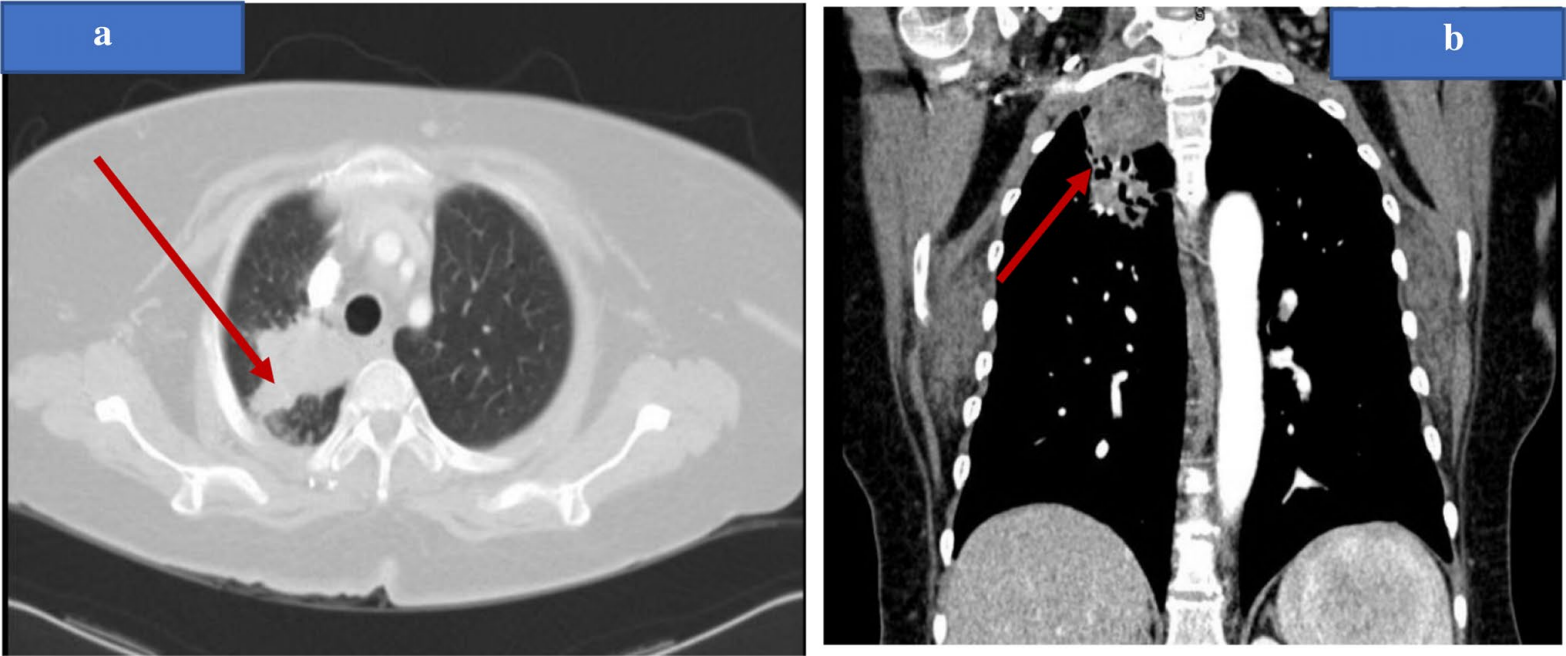
**a**, **b** Chest CT showing a well-defined right apical mass lesion with pleural invasion and possible mediastinal extension, suggestive of Pancoast tumor (red arrow)

A 58-year-old female patient, with 20 pack-year smoking history, and type 2 Diabetes mellitus for 2 years. She presented in 2015, with an incidental right apical lung mass suggestive of Pancoast tumor as demonstrated on her chest CT (Fig. 1a, b) following abnormal chest X-ray. The patient refused further investigations at the time. 3 years later, she was admitted to with sepsis secondary to acute cholecystitis.

**Fig. 2:**
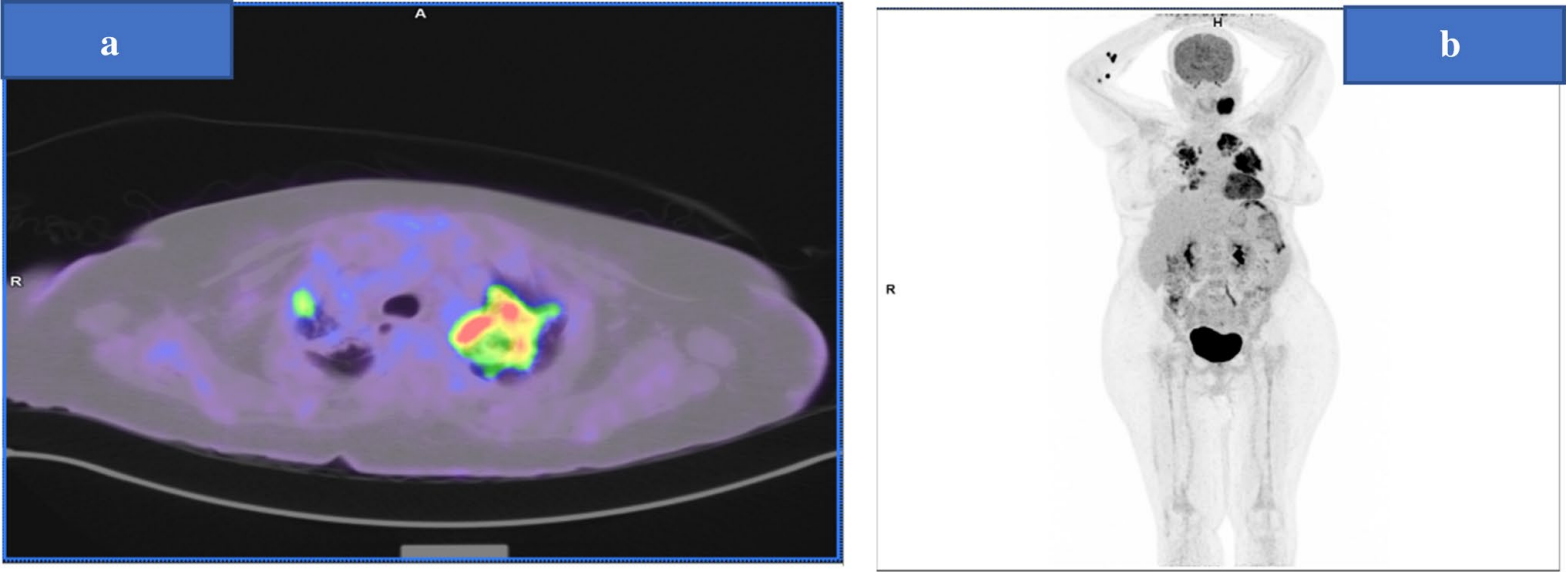
**a** Fused FDG PET-CT image with lung window showing intense uptake bilaterally more prominent in the left apical mass. **b** maximum intensity projection representation of whole body FDG distribution showing metastatic pulmonary adenocarcinoma (stage IV)—note FDG avidity is more on left side

Hospital course was complicated by left anterior cerebral artery (ACA) stroke. During the admission, further workup of her lung mass was pursued through biopsy from a left cervical lymph node, which affirmed the diagnosis of metastatic pulmonary adenocarcinoma (stage IV) (Fig. 2a, b).

**Fig. 3:**
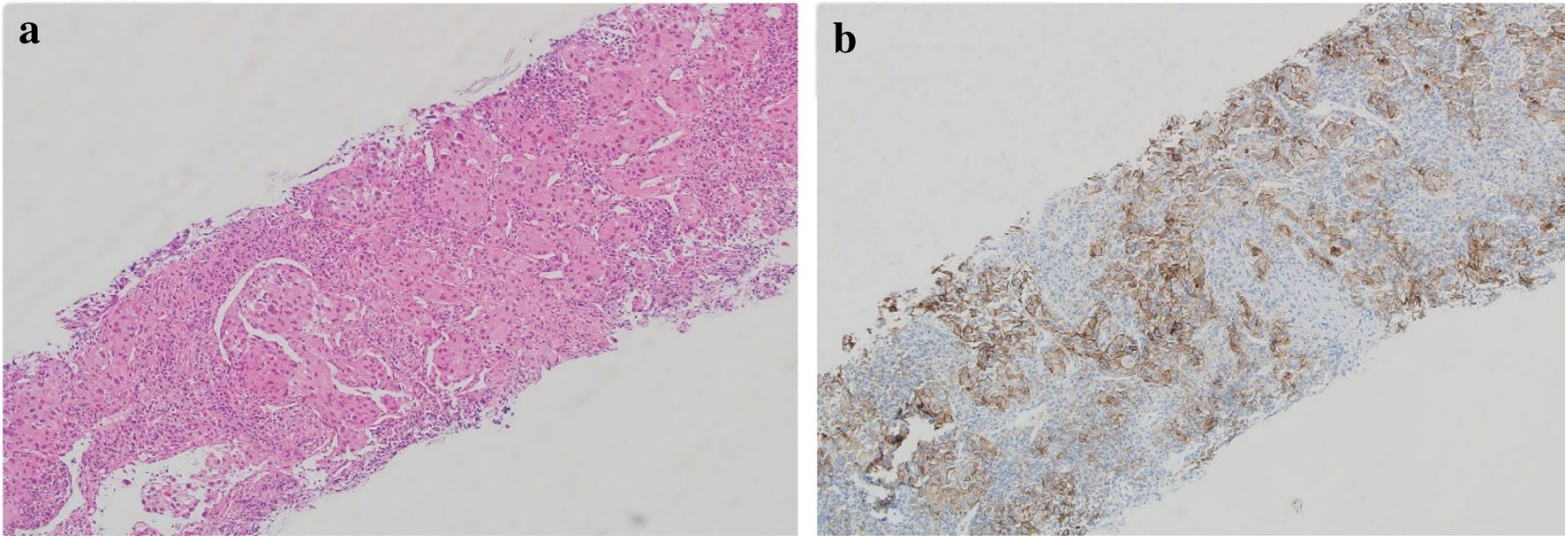
**a** H&E lymph node is extensively infiltrated by nests and sheets of large malignant cells with large irregular-shaped nuclei, prominent nucleoli and abundant eosinophilic cytoplasm. Rare scattered anaplastic cells are also present. **b** immunohistochemistry showing PDL 1 is strongly positive in 95% of cells

Immunohistochemistry (IHC) report was strongly positive for PD-L1 in 95% of the tumor cells (Fig. 3a, b), negative ALK gene rearrangement, and no EGFR mutation was detected.

**Fig. 4:**
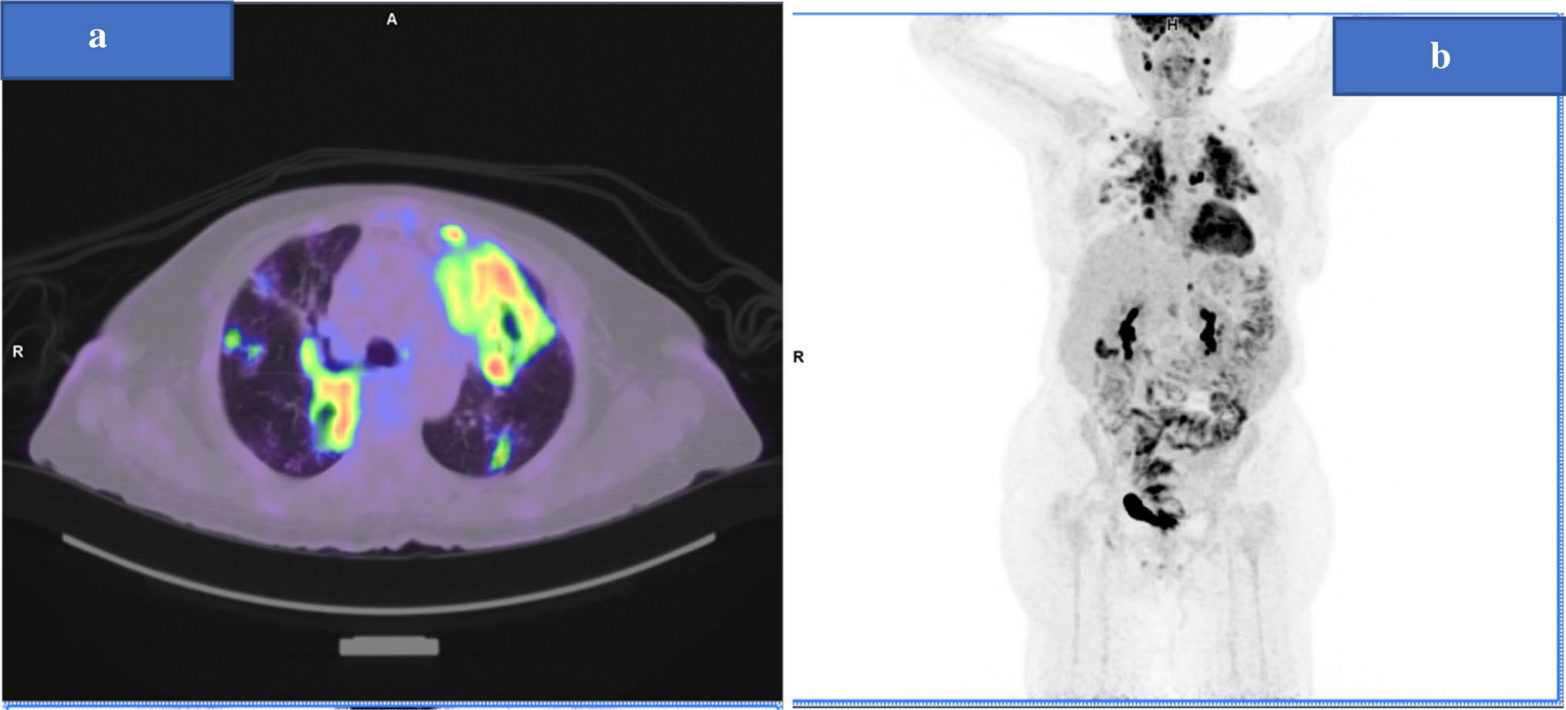
**a**, **b** PET CT (lung window) following six cycles of pembrolizumab showed mixed response with overall moderate progression

Later on, the patient was started on immunotherapy pembrolizumab monotherapy, 200 mg every 3 weeks, as first-line treatment. PET CT following six cycles of pembrolizumab showed mixed response with overall moderate progression (Fig. 4a, b).

**Fig. 5:**
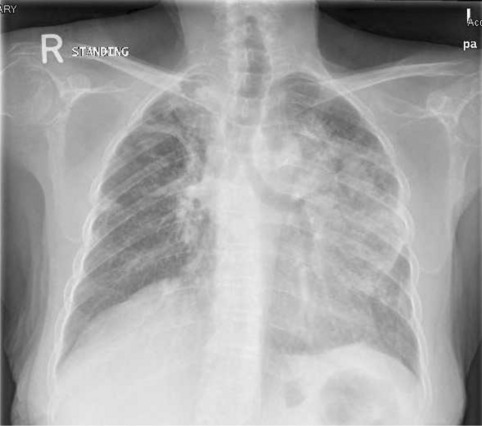
Chest XR showing large opacity noticed in the left mid and lower lung zones and Right upper zonal nodular and reticular shadowing

On 29th July 2019, she presented with a 1-week history of productive cough, high-grade fever, tachycardia, and low oxygen saturation (Temp: 39.2, HR: 130, SPO2: 91% on room air). Chest X-ray revealed a significant opacity in the left mid and lower lung zones (Fig. 5).

**Fig. 6:**
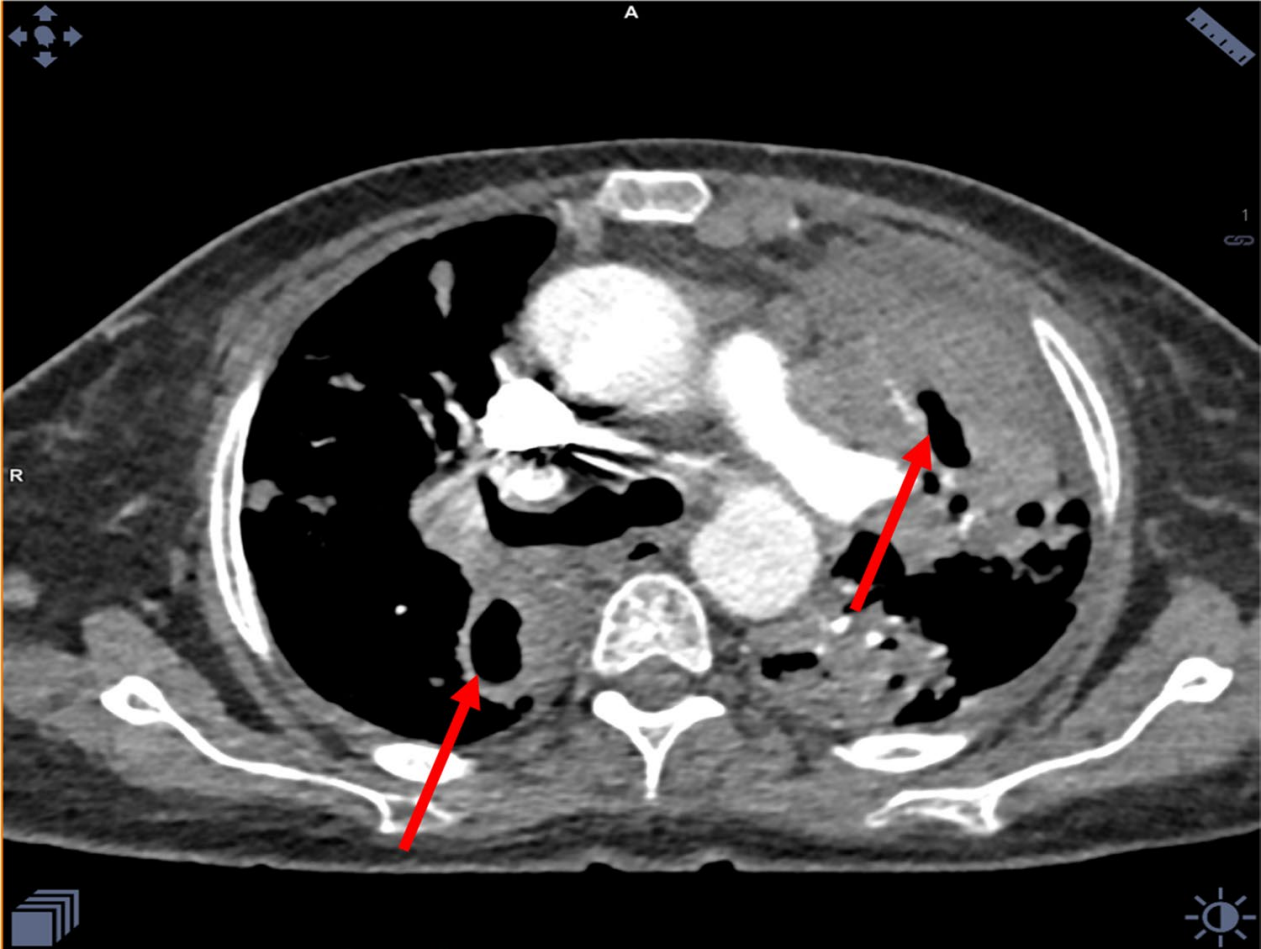
CT chest angiogram (lung window) showing consolidation with cavitary lesion bilaterally (red arrow)

CT angiogram was done and showed left-sided large pulmonary consolidative mass lesion and areas of cavitation. (Fig. 6).

She was treated with antibiotics as community-acquired pneumonia; however, she continued to spike fever. The initial microbiological workup was negative. Bronchoscopy and bronchoalveolar lavage (BAL) showed positive AFB smear and TB PCR (rifampin resistance not detected). No workup for pulmonary tuberculosis was done prior to initiation of ICPs.

The patient home medications prior to the last hospital admission included; atorvastatin 40 mg oral tablet once daily, aspirin 100 mg oral tablet once daily and vitamin D2 capsules oral once weekly. During her course of immunotherapy, she did not experience any immune-related adverse events (irAEs) requiring prednisone prior to development of TB.

She was started on anti-TB medications (RIFA four). Further history revealed that she had sick contact with active TB patient 10 years ago, but there was no documentation of latent TB or previous TB infection. Her HIV status was negative. Sputum AFB smear found positive and cleared 6 weeks following anti-TB medications.

**Fig. 7:**
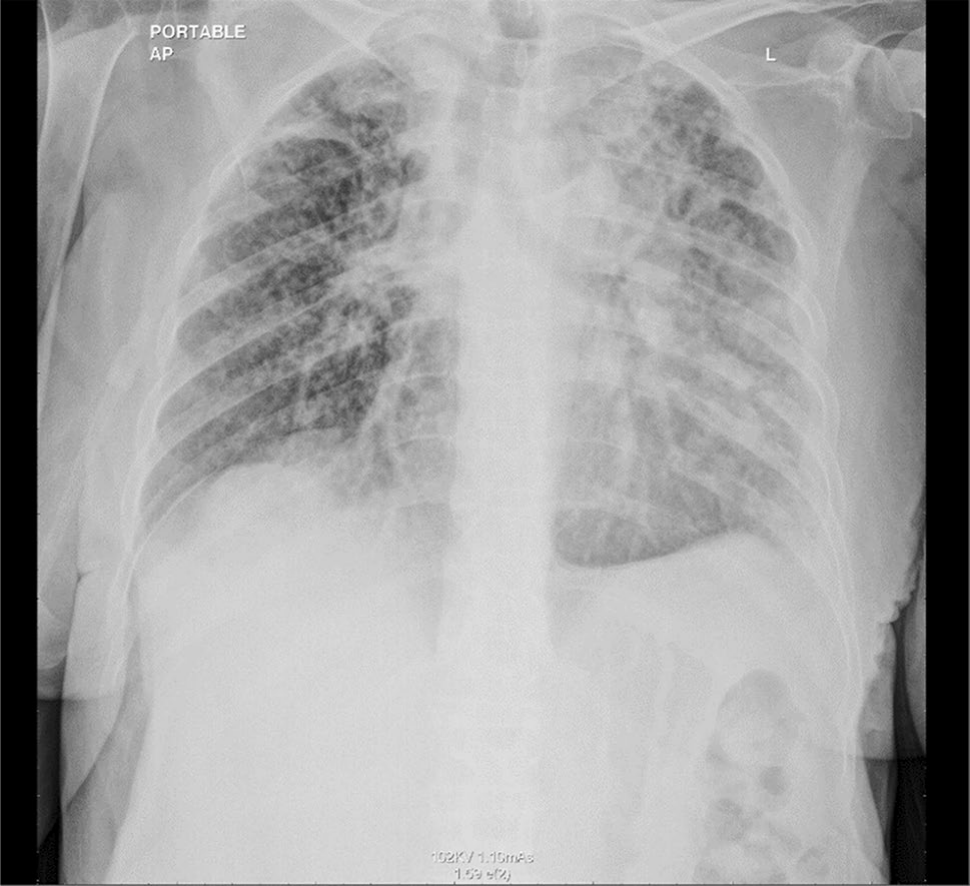
Chest X-ray showed new bilateral reticular and nodular pulmonary infiltrates.

2 weeks later, chest X-ray showed new bilateral reticular, and nodular pulmonary infiltrates (Fig. 7).

**Fig. 8:**
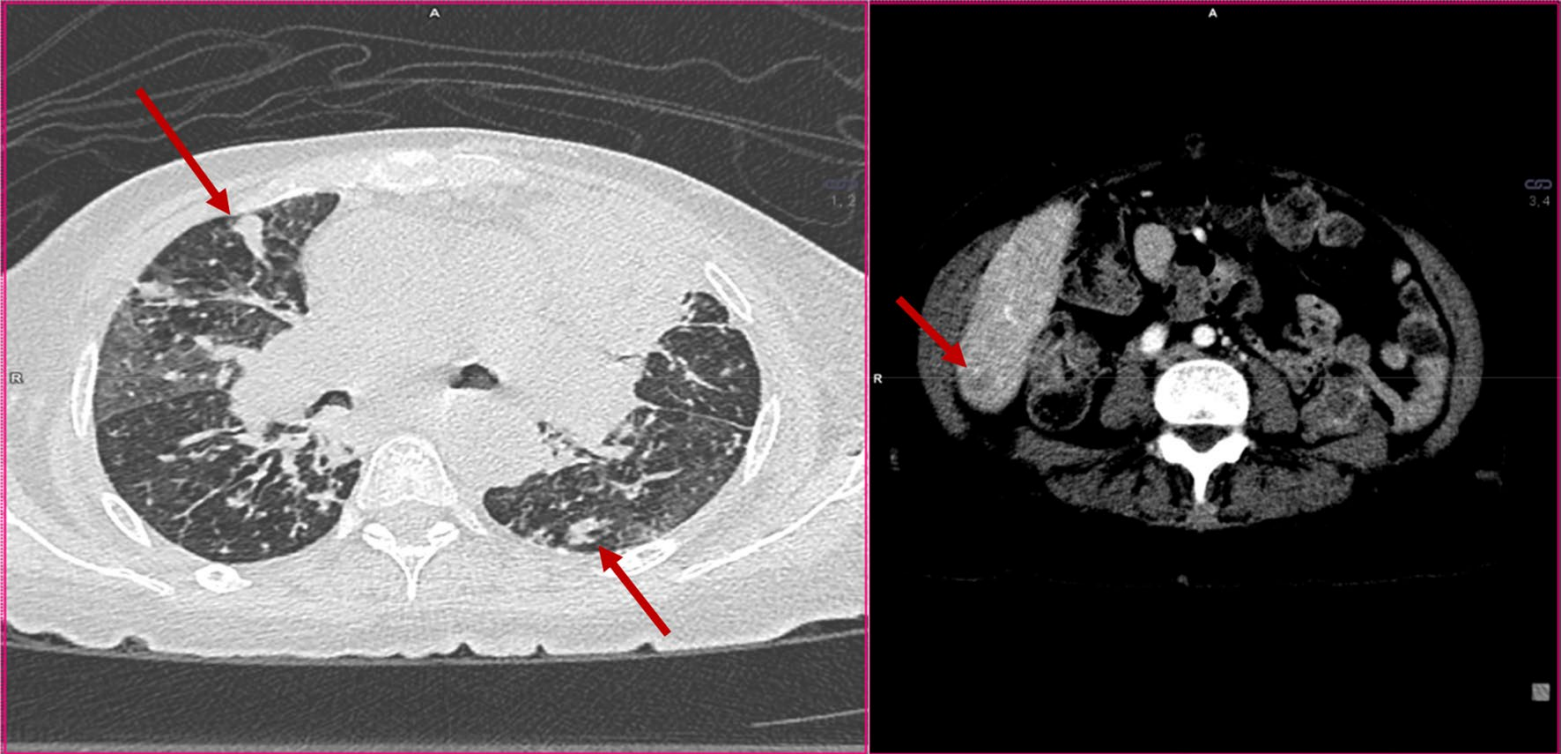
CT chest confirmed progression of the malignant disease, with innumerable bilateral nodular lesions, and new hypodense lesion in segment 6 of the liver.

CT chest was done and confirmed the progression of the disease, with innumerable bilateral lesions and new lesion in segment 6 of the liver (Fig. 8).

Patient was subsequently started chemotherapy with carboplatin and pemetrexed as second line. Repeated PET CT after four cycles of second-line chemotherapy showed Good therapeutic response with near-complete remission of lung, liver, spleen, and lymph nodal involvements (Supplementary figure 9).

The patient remained on chemotherapy and anti-TB medications for a proposed 9-month duration.

## Discussion

TB reactivation is an established adverse effect attributed to many anti-cancer biological agents and with TNF-α inhibitors as well [7, 8]. The incidence of TB reactivation in cancer patients is higher in hematological malignancies compared to solid tumors. While, among solid tumors, the highest incidence of TB reactivation was reported in lung cancer followed by gastric cancer, breast cancer, liver cancer, and colon cancer, respectively [9].

With the expanding use of immune checkpoint inhibitors for the management of cancer, infectious complications of immune checkpoint inhibitors became an emerging adverse effect of these agents, including TB reactivation [10].

The majority of patients infected with tuberculosis will develop a latency state with no signs of disease, with approximately, up to ten percent of those patients may develop active tuberculosis infection [11]. Containments of the infection are mediated by cytokines and the interaction between macrophages and T lymphocytes (CD4 and CD8) [12]. Immunocompromised status including, HIV, organ transplanted patients, and patients receiving immunosuppressive therapy is one of the most critical risk factors for TB reactivation [13].

The exact mechanism of TB reactivation following treatment with these agents remains unclear, with further studies is warranted. However, few preclinical studies in MTB infected PD-1-deficient mice and PD-1 blocked humans describe an increase in the IFN-α production by CD4 T cells which promote more bacterial replication and tissue destruction [6, 14, 15].

Furthermore, the role of (PD-L1/PD-1) pathway has been studied which has demonstrated its effect on *M. tuberculosis* infection; In mice model, PD-1 deficiency showed significant sensitivity to *M. tuberculosis* infection and high bacillary load after exposure to aerosol infection with *M. tuberculosis*. PD-1-deficient mice also showed dramatic survival reduction and lung tissue was found to be severely necrotic and inflamed in comparison to the control group [16]. On the other hand, the data about (PD-L1/PD-1) pathway and its role in the cytolytic activity of *T. lymphocytes* in humans is diversely contradictory [17]. However, multiple reports highlighted the reactivation of pulmonary tuberculosis infection after the use of PD-1 inhibitors [10, 15, 26, 27, 18–25].

In this paper, ICIs associated MTB infection was extensively searched by expediting all the reported cases through PubMed up to September 2019, with no language restriction applied. In general, 15 reported cases were identified retrieved from 12 articles [10, 15, 26, 27, 18–25], in addition to our case (Table 1). Data showed that all the patients were either Caucasians or Asians, aged from 49 to 87 years and with male predominance.

With respect to their oncological diagnosis, five cases had metastatic non-small cell lung cancer (NSCLC), six cases had metastatic melanoma, two cases had metastatic head and neck squamous cell carcinoma (HNSCC), one case had Hodgkin lymphoma and one case had metastatic Merkel carcinoma.

For the ICIs, eight cases were on nivolumab, six cases were on pembrolizumab, and only one case was on atezolizumab. The time to diagnosis varied among patients and ranged between 4 and 36 weeks. In all patients, no latent TB testing (LTBT) before immunotherapy was done, and it was not clear whether TB infection is primary or secondary to latent TB reactivation. TB was microbiologically confirmed in all cases and followed by anti-TB drugs initiation. ICIs were maintained in three cases and discontinued or temporarily suspended in the remaining patients.

The time to diagnosis of TB in the current case occurred after six cycles of Pembrolizumab. TB was confirmed microbiologically by PCR and AFB. The patient received her BCG vaccine as part of the local child immunization program. Our case gave a history of sick contact with a patient with active TB infection 10 years ago, but there was no documentation of latent TB or previous TB infection prior to initiation of ICPs. The mixed response noted on 14th July 2019 PET CT (Fig. 4) was not perceived as pseudoprogression-like phenomenon as overt disease progression was confirmed by 26th August 2019 CT chest and abdomen as illustrated (Fig. 8).

ICIs were not resumed in our case and carboplatin plus pemetrexed was initiated instead, as second-line chemotherapy. None of the previously reported cases has used the traditional chemotherapy as a subsequent therapy to immunotherapy; nonetheless, the outcome of TB in patients receiving cytotoxic chemotherapy for malignancies have been reported in two retrospective studies in South Korea and Japan [28, 29]. In both studies, concurrent chemotherapy was found to be effective and safe for treating cancer patients with active MTB.

In a recent Meta-analysis including United States cancer patients, the risk of active TB was 41/100,000 [30], however, it is significantly higher in high prevalence areas such as South Korea with 3.07/1000 in patients with cancer [31]. It is worth mentioning that the global prevalence of latent TB infection in 2014 was estimated to be 23.0%, while the estimate for WHO Eastern Mediterranean Region which includes the state of Qatar was 16.3 [13.4–20.5] [32].

In 2012, the incidence of tuberculosis in Qatar was 41/100,000. The majority of infected patients (90%) was non-national males [33]. Whereas, pulmonary tuberculosis represents around 46% of active tuberculosis infection [34].

## Conclusion

To our knowledge, this is the first reported case from the Arab and the Middle East region; it reinforces the previous observations of the association between ICIs administration and the development of MTB. Nevertheless, furthers studies in the clinical setting are necessary to establish the exact mechanism involved in this association. Oncologists’ awareness and prompt recognition of this potential hazardous consequence are essential. Since there is no clear evidence whether LTBT prior PD-1/PD targeted immunotherapy is required, targeted LTBT before starting ICIs immunotherapy with TB chemoprophylaxis; yet to be explored, particularly in the regions where the MTB prevalence is high.

## Electronic supplementary material

Below is the link to the electronic supplementary material.

Supplementary file1 (DOCX 2364 kb)

## Data Availability

The datasets used and/or analyzed during the current study are available from the corresponding author on reasonable request.
