# Supplementary material for: Tuberculosis following programmed cell death receptor-1 (PD-1) inhibitor in a patient with non-small cell lung cancer. Case report and literature review
Source: Cancer Immunol Immunother. 2020 Oct 17;70(4):935–44. doi: 10.1007/s00262-020-02726-1 (PMC7979647; doi:10.1007/s00262-020-02726-1)
Supplement: Supplementary file 1 — Supplementary file1 (DOCX 2364 kb) [file 262_2020_2726_MOESM1_ESM.docx]

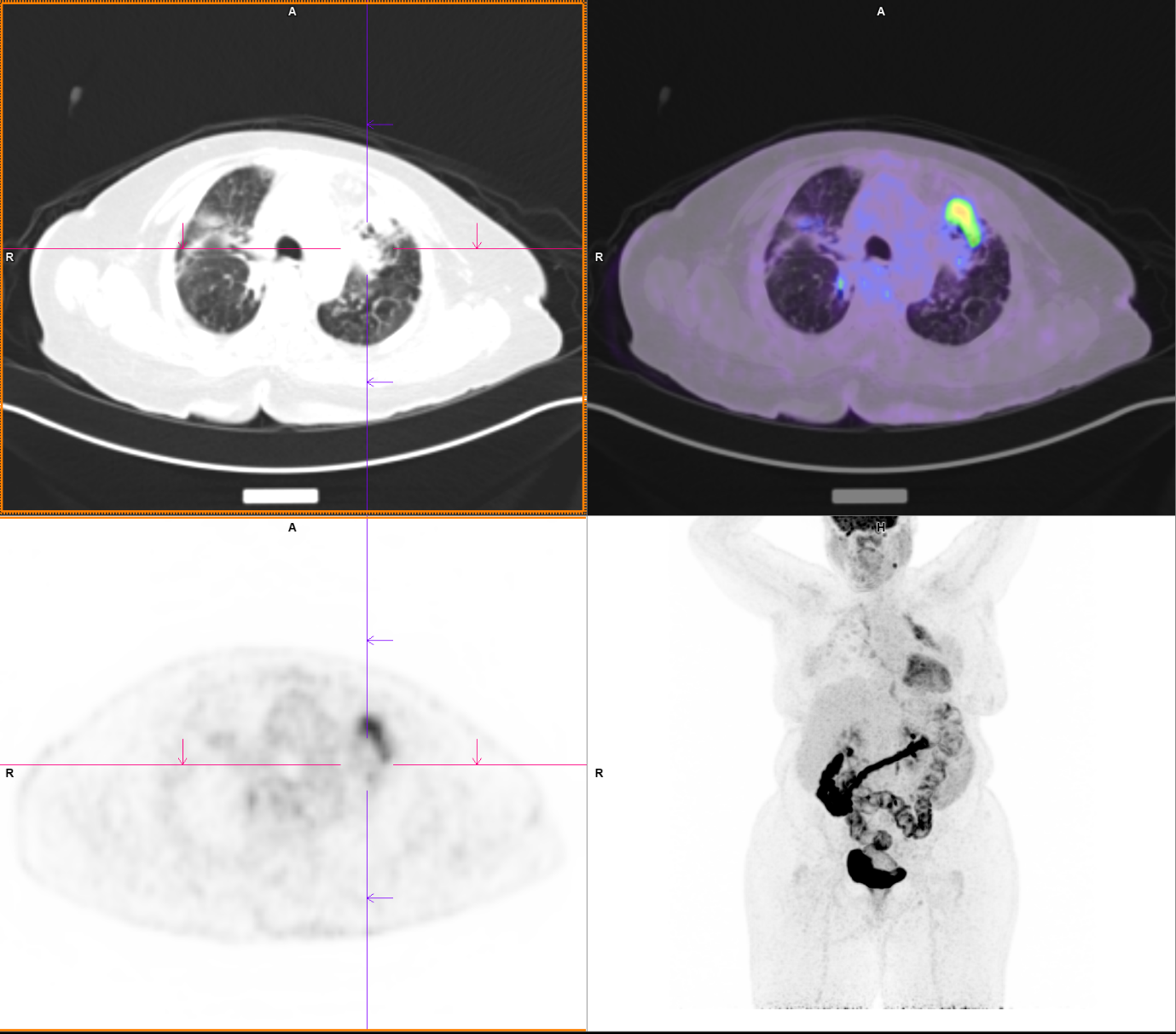

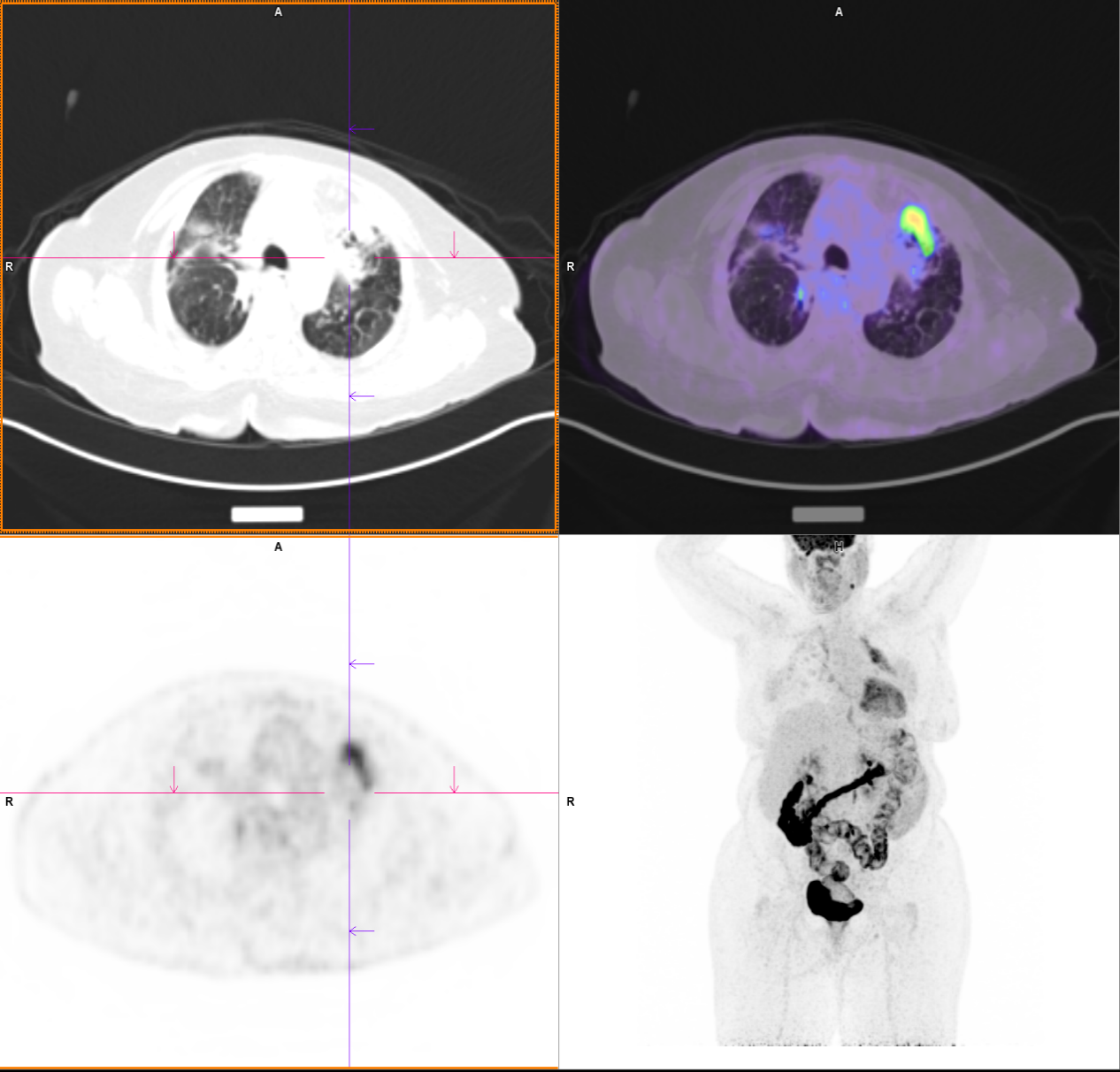
Figure 9 : PET CT following 4 cycles of pemetrexed and carboplatin chemotherapy showed near complete response.
